# Supplementary material for: Polydopamine-Assisted Rapid One-Step Immobilization of L-Arginine in Capillary as Immobilized Chiral Ligands for Enantioseparation of Dansyl Amino Acids by Chiral Ligand Exchange Capillary Electrochromatography
Source: Molecules. 2021 Mar 23;26(6):1800. doi: 10.3390/molecules26061800 (PMC8004743; doi:10.3390/molecules26061800)
Supplement: Supplementary file 1 [file molecules-26-01800-s001.pdf]

Supplementary data

Polydopamine-assisted rapid one-step immobilization of L-arginine in capillary as immobilized chiral ligands for enantioseparation of dansyl amino acids by chiral ligand exchange capillary electrochromatography

Yuanqi Gui [1,†], Baian Ji [1,†], Gaoyi Yi [1], Xiuju Li [2,*], Kailian Zhang [1] and Qifeng Fu [1,*]

[1] School of Pharmacy, Southwest Medical University, Luzhou 646000, China; guiyuanqi1997@163.com (Y.G.); jibaian1997@163.com (B.J.); yigaoyi169216@163.com (G.Y.) zkl66@swmu.edu.cn (K.Z.)
[2] School of Pharmacy, Tongren Polytechnic College, Tongren 554300, China
* Correspondence: lxj_trzy@163.com (X.L.); fuqifeng1990@163.com (Q.F.); Tel.: +86-856-6909046 (X.L.); +86-830-3161291 (Q.F.)
† These authors contributed equally to this work.

Table of contents

1. Influence of dopamine concentration and the molar ratio of dopamine to L-Arg on the enantioseparation
2. Effect of reaction temperature, reaction time on the enantioseparation and theoretical plate numbers
3. Digital photographs of PDA/L-Arg hybrid coated quartz plates fabricated under different reaction temperature or different reaction time
4. Influence of applied voltages on the enantioseparation
5. Continuous electrophoresis operation tests
6. Comparison of enantioseparation performance for coated and bare capillaries
7. Comparison of enantioseparation performance for different CLE-CEC systems
8. Relative standard deviations of the retention time of Dns-D, L-AAs in intra-day and inter-day and column-to-column
9. Quantitative features of the CLE-CEC method for determination of D-Glu and L-Glu

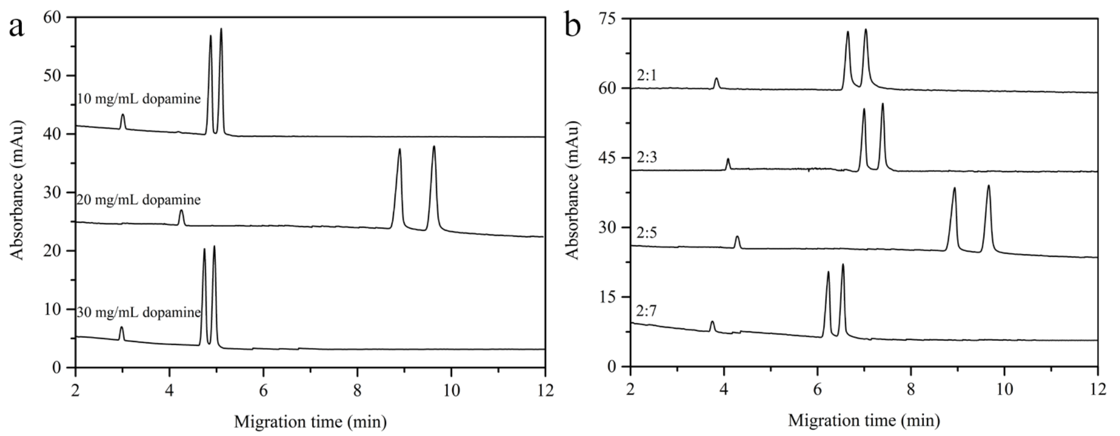

Figure S1. (a) Electropherograms of Dns-D, L-Ala on PDA/L-Arg@capillary fabricated by using 250 mM L-Arg and different concentrations of dopamine. (b) Electropherograms of Dns-D, L-Ala on PDA/L-Arg@capillary fabricated by using different molar ratio of dopamine to L-Arg. All conditions are the same as Figure 5.

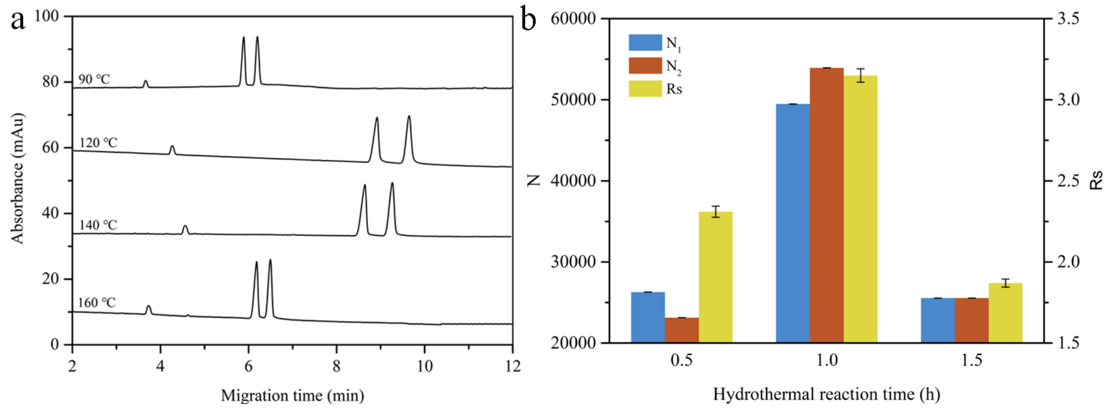

Figure S2. (a) Electropherograms of Dns-D, L-Ala on PDA/L-Arg@capillary fabricated under different reaction temperature. (b) Influence of hydrothermal reaction time on the resolutions and theoretical plate numbers of Dns-D, L-Ala. All the CE experimental conditions are the same as Figure 5.

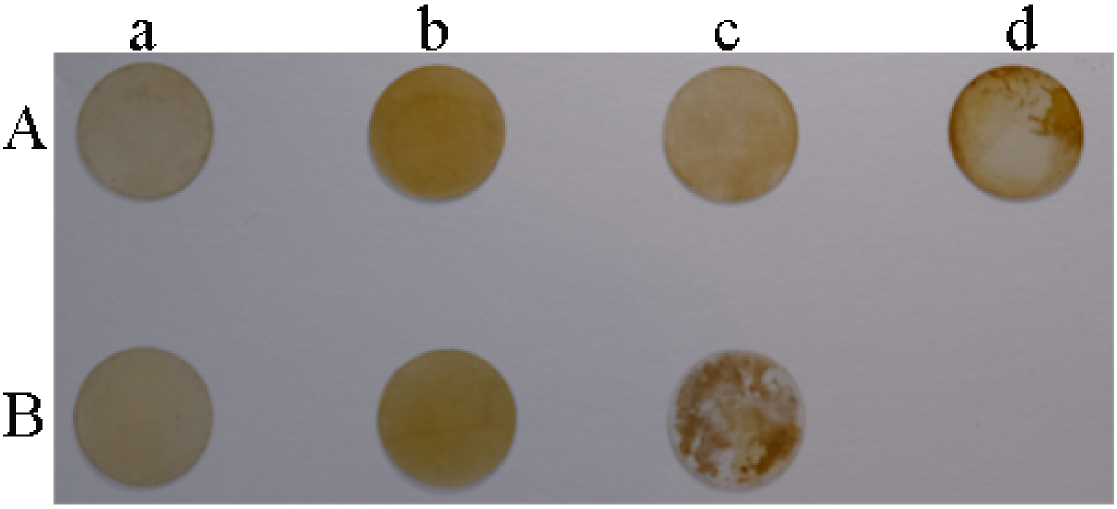

Figure S3. (A) Digital photographs of PDA/L-Arg coated quartz plates fabricated under reaction time of 1 h with different reaction temperature of 90 °C (a), 120 °C (b), 140 °C (c), 160 °C (d). (B) Digital photographs of PDA/L-Arg coated quartz plates fabricated under 120 °C with different reaction times of 30 min (a), 1 h (b), 1.5 h (c).

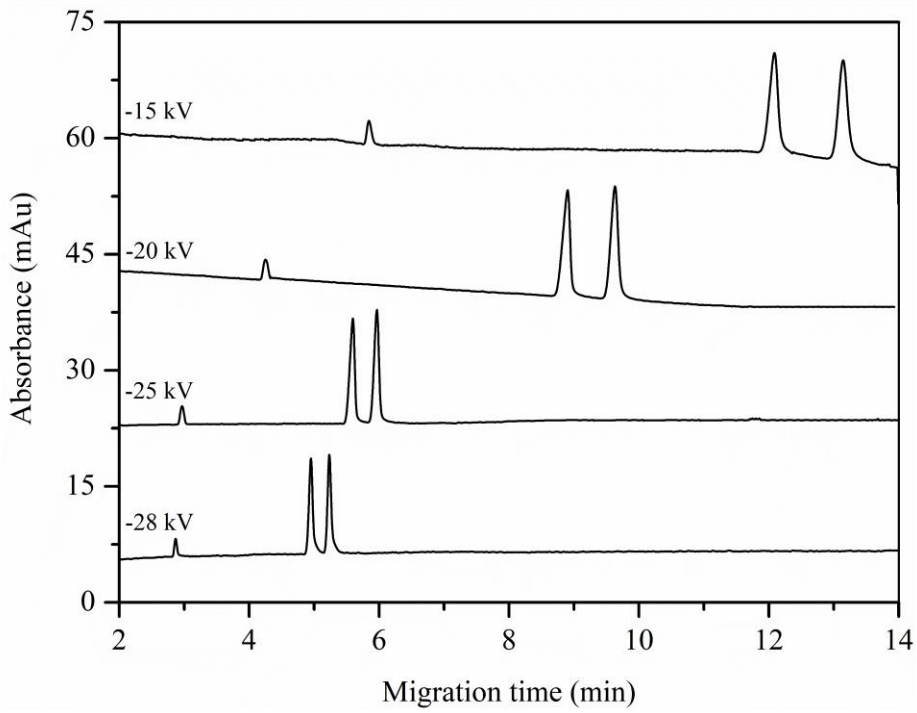

Figure S4. Electropherograms of Dns-D, L-Ala on PDA/L-Arg@capillary at different voltages ranged from -15.0 kV to -28.0 kV. All the CE experimental conditions are the same as Figure 5.

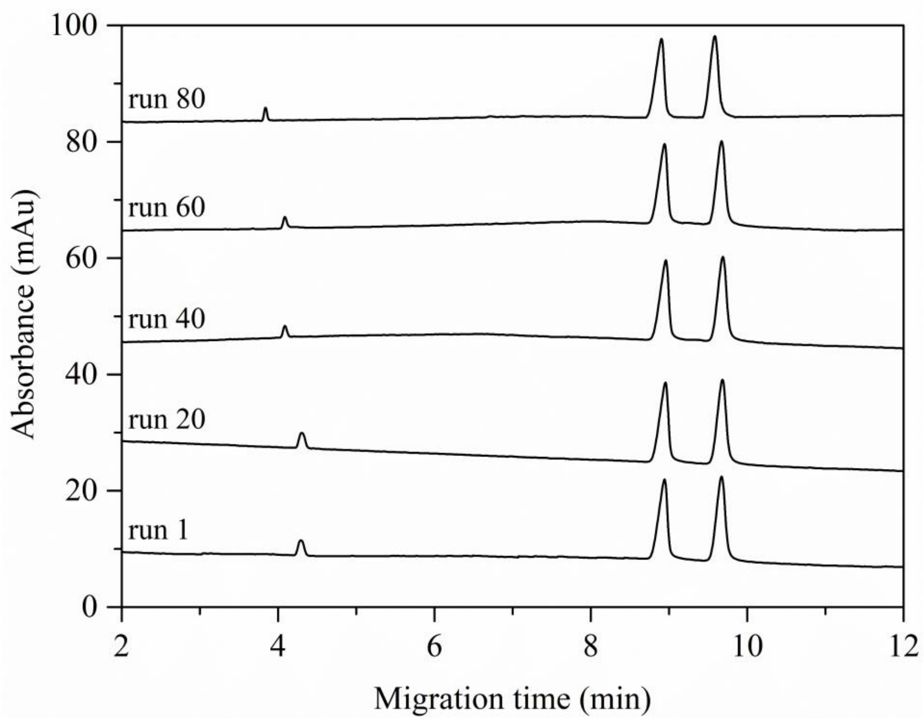

Figure S5. Electropherograms of Dns-D, L-Ala after 80 consecutive runs on PDA/L-Arg@capillary. All the CE experimental conditions are the same as Figure 5.

Table S1. Enantioseparation performances of Dns-D, L-AAs with PDA/L-Arg@capillary and bare capillary.

Dns-D, L-AAs	[a] Bare capillary			[b] PDA/L-Arg@capillary		
**Dns-D, L-AAs**	[c] **Rs**	[d] **t_D / min**	[e] **t_L / min**	[c] **Rs**	[d] **t_D / min**	[e] **t_L / min**
Dns-D,L-Asp	0.74±0.09	4.05±0.10	4.18±0.20	3.16±0.10	8.91±0.10	9.34±0.10
Dns-D,L-Ala	1.22±0.03	4.87±0.06	5.05±0.10	3.15±0.03	6.93±0.30	7.33±0.07
Dns-D,L-Ser	1.54± 0.04	4.63±0.71	4.92±0.50	5.28±0.04	8.23±0.04	9.03±0.07
Dns-D,L-Gln	0.66±0.04	4.71±0.53	4.85±0.47	2.98±0.05	7.51±0.09	7.96±0.20
Dns-D,L-Met	1.29±0.02	4.98±0.80	5.19±0.39	3.14±0.20	7.57±1.20	8.20±0.20
Dns-D,L-Ile	1.03±0.01	4.45±0.70	4.56±0.80	2.39±0.03	6.99±0.80	7.34±0.20
Dns-D,L-Asn	1.47±0.04	5.22±0.45	5.41±0.60	2.16±0.05	10.02±0.08	10.68±0.15
Dns-D,L-Thr	0.75±0.03	4.30±0.5	4.38±0.09	1.82±0.01	6.44±0.06	6.74±0.08
Dns-D,L-Leu	0.39±0.01	4.34±0.09	4.38±0.08	1.07±0.09	6.21±0.20	6.38±0.10
Dns-D,L-Pro	0	2.98±0.10	2.98±0.10	0.78±0.10	5.24±0.40	5.38±0.65
Dns-D,L-Phe	0	4.69±0.07	4.69±0.07	0.66±0.10	7.12±0.30	7.27±0.20

[a] CLE-CEC conditions were same as that in Figure 5 with PDA/L-Arg@capillary;

[b] CLE-CE conditions were same as that in Figure 5 with bare capillary;

[c] chiral resolution of Dns-D, L-AAs;

[d] migration time of Dns-D-AAs;

[e] migration time of Dns-L-AAs.

Table S2. Comparison with enantioseparation of D, L-AAs using different CLE-CEC systems.

**No.**	**Types of Capillary**	**fabrication time**	**Organic solvent**	**Rs>1.5**	**Rs<1.5**	**migration time**	**Reference**
1	Continuous bed capillary	>12 h	No	2 pairs	7 pairs	7-27 min	[34]
2	Silica monolithic capillary	>2 days	70% ACN	12 pairs	-*	5-16 min	[33]
3	Silica monolithic capillary	>1 week	70% ACN	2 pairs	2 pairs	22-24 min	[24]
4	Coated capillary	>4 days	No	5 pairs	5 pairs	6-22 min	[8]
5	Coated capillary	>2 days	No	7 pairs	5 pairs	20-67 min	[7]
6	Coated capillary	1 h	No	8 pairs	3 pairs	5-11 min	This study

*Not mentioned

Table S3. Relative standard deviations (RSD%) of the retention time of Dns-D, L-AAs in intra-day and inter-day and column-to-column. [a]

Dns-D,L-AAs	Intra-day (n=5)		Inter-day (n=3)		column-to-column (n=3)	
	RSD_{t_1} [b]	RSD_{t_2} [c]	RSD_{t_1} [b]	RSD_{t_2} [c]	RSD_{t_1} [b]	RSD_{t_2} [c]
Dns-D,L-Asp	1.20	2.84	2.00	3.35	4.90	4.15
Dns-D,L-Ala	1.96	2.47	3.60	2.90	3.89	5.70
Dns-D,L-Ser	1.90	1.30	2.63	2.29	5.76	5.16

[a] CLE-CEC conditions were same as that in Figure 5

[b] migration time of Dns-D-AAs

[c] migration time of Dns-L-AAs

Table S4. Quantitative results of the CLE-CEC system for Dns-D-Glu and Dns-L-Glu. [a]

Dns-D,L-Glu	Linear relationship [b]	r^2	Linearity range (μg/mL)	LOD (μg/mL)	LOQ (μg/mL)
Dns-D-Glu	Y=0.0721*X+0.3375	0.9985	15-800	15	50
Dns-L-Glu	Y=0.0706*X+0.5172	0.9980	15-800	15	50

[a] CLE-CEC conditions were same as in Figure 5;

[b] Y represents corrected peak area; X represents the concentration of Dns-D-Glu or Dns-L-Glu.
